# Root-Specific Signal Modules Mediating Abiotic Stress Tolerance in Fruit Crops

**DOI:** 10.3390/plants15030363

**Published:** 2026-01-24

**Authors:** Lili Xu, Xianpu Wang

**Affiliations:** Agricultural College, Shihezi University, Shihezi 832003, China; xull_agr@shzu.edu.cn

**Keywords:** fruit crops, root, abiotic stress, signal modules

## Abstract

Sustained abiotic stress severely impairs fruit crop growth and development. As plants’ primary environmental sensing organ, fruit tree roots experience disrupted morphogenesis and physiological functions, reducing yield, lowering fruit quality, and threatening orchard ecosystem stability. Abiotic stress is diverse: water deficit from drought, extreme temperature fluctuations, and salinization-induced ion imbalance, heavy metal accumulation, or nutrient disorders. Its complexity requires synergistic and crosstalk regulation of multiple root-specific signaling modules and pathways in root stress perception and transduction. When responding to stress, roots activate hormone, reactive oxygen species (ROS), and calcium ion (Ca^2+^) signaling. These pathways mediate early stress recognition and regulate downstream gene expression and physiological metabolic reprogramming via transcription factors (TFs) and other regulators, determining stress tolerance and adaptability. Using typical abiotic stresses as models, this review outlines the composition, activation mechanisms, specificity, and synergistic effects of root-specific signaling modules/pathways, along with modern biotechnologies for decoding these modules and current research limitations, aiming to reveal the root signal network’s integration mode.

## 1. Introduction

Fruits are indispensable components of a healthy human diet, serving as natural reservoirs of phytochemicals, essential nutrients, and dietary fiber [1]. Beyond basic nutrition, many fruit-derived natural compounds exhibit diverse biological activities; for example, resveratrol found in grapes and berries can alleviate inflammation both acutely and chronically [2,3] and even reduce the risk of certain cancers [4]. Economically, the global fruit industry constitutes a critical sector of agricultural production, supporting the livelihoods of millions of farmers and contributing significantly to national economies through domestic consumption and international trade. Fruits such as apples, grapes, citrus, and strawberries not only meet consumer demand for fresh produce but also serve as raw materials for processed products including juices, jams, wines, and functional foods, creating extensive value chains [5,6,7,8]. However, the sustainability and profitability of fruit cultivation are increasingly threatened by abiotic stresses intensified by global climate change. These stresses—encompassing drought, saline–alkali, extreme temperatures, flooding, heavy metal contamination, and nutrient deficiency—disrupt fruit crop growth and development at multiple stages, leading to reduced yields, compromised fruit quality (e.g., poor coloring, reduced sugar content), increased susceptibility to secondary stresses, and elevated production costs [9,10,11]. In severe cases, widespread crop failure can occur, threatening global fruit supply security, undermining orchard ecosystem stability, and endangering the economic viability of fruit farming. Addressing these production challenges by enhancing abiotic stress tolerance in fruit crops has become an urgent priority for agricultural research and industry development.

As the primary organ for perceiving environmental cues and acquiring water and nutrients, plant roots play an irreplaceable role in mediating fruit crop adaptation to abiotic stress. Unlike above-ground organs, roots are in direct contact with the soil environment, making them the first line of defense against soil-borne stresses. Root exhibits remarkable plasticity, adapting to adverse conditions through integrated morphological, anatomical, and physiological adjustments to expand the absorption surface area. They also secrete large amounts of organic acids (e.g., citric acid) to dissolve insoluble soil elements and activate high-affinity ion transporters, thereby promoting nutrient uptake [12]. The growth and functional adaptation of fruit tree roots are modulated by a complex interplay of internal and external factors. External stressors directly alter internal regulatory networks, reshaping root architecture and function to optimize resource acquisition and stress resistance [13]. Notably, fruit trees, as woody plants characterized by long growth cycles (3–8 years from germination to flowering) and complex heterozygous, often polyploid genetic backgrounds, differ fundamentally from herbaceous plants in their root stress-resistant signaling mechanisms [14]. Compared with herbaceous plants that have short life cycles, simple genetic backgrounds, and easy genetic transformation, fruit tree roots have evolved unique signaling regulatory features to cope with long-term abiotic stress exposure [15]. For instance, woody roots possess enhanced lignification and suberization capabilities to form physical barriers against stress, and their signaling networks involve more persistent epigenetic regulation to maintain stress memory across growth cycles, with these traits being less prominent in herbaceous roots [16]. These core differences arise from the distinct evolutionary strategies of woody and herbaceous plants, with fruit trees prioritizing long-term stress adaptation and survival, while herbaceous plants focus on rapid growth and generational renewal.

The ability of fruit tree roots to cope with abiotic stress is underpinned by intricate molecular regulatory networks that enable precise sensing, signal transduction, and response activation. At the molecular level, stress adaptation involves multi-layered regulation, including signal perception by membrane-localized receptors, activation of second messenger systems, transduction through conserved signaling cascades, and transcription factor-mediated gene expression reprogramming. Additionally, epigenetic modifications such as DNA methylation, histone modifications, and non-coding RNAs fine-tune gene expression patterns to enhance stress adaptation without altering the underlying DNA sequence. These molecular regulatory networks integrate internal developmental signals and external environmental cues, allowing roots to mount coordinated responses to diverse abiotic stresses. Elucidating the composition, activation mechanisms, and crosstalk of these molecular networks is not only fundamental to advancing our understanding of plant–environment interactions but also provides valuable molecular targets for biotechnological improvement of fruit crop stress tolerance [17,18,19].

Given the economic and nutritional importance of fruit crops, the escalating threats posed by abiotic stresses, and the central role of roots in stress adaptation, comprehensive research on root-specific regulatory mechanisms is essential. This review aims to synthesize current knowledge on root-mediated abiotic stress tolerance in fruit crops, focusing on the core regulatory frameworks rather than redundant details of specific pathways or species-specific mechanisms. We systematically examine the critical role of roots in stress response, summarize the general principles of molecular regulation underlying root adaptation, and highlight the significance of decoding root-specific signaling modules. Additionally, we discuss the applications of modern biotechnologies in dissecting root stress networks and address current research gaps and future directions. By providing a comprehensive conceptual framework, this review seeks to lay the foundation for further research on root-mediated stress tolerance and support the development of stress-resistant fruit crop varieties and sustainable cultivation practices, ultimately ensuring the stability and productivity of the global fruit industry.

## 2. Roots of Fruit Crops

**Figure 1:**
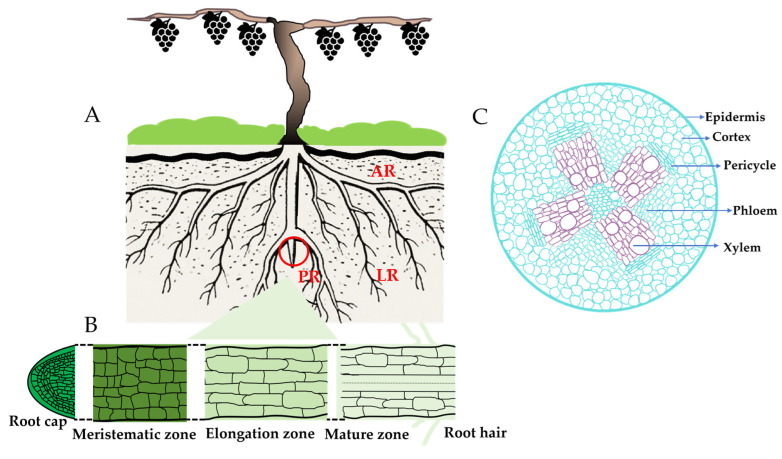
Schematic diagram of grapevine root architecture and root anatomical structure. (**A**) Overall root system architecture of apple at late developmental stage. Abbreviations: PR, primary root; LR, lateral root; AR, adventitious root. The circle indicates the local area corresponding to the root longitudinal section below. (**B**) Longitudinal section of apple primary root, showing four functional zones: Root cap (protective structure at root tip), Meristematic zone (cell division region), Elongation zone (cell elongation region), and Mature zone (with differentiated Root hair for nutrient absorption). (**C**) Transverse section of the mature zone in apple root, showing the anatomical structure from outer to inner: Epidermis (outermost layer), Cortex (parenchyma tissue), Pericycle (lateral root initiation layer), xylem and phloem (nutrient transport structure).

During the long-term evolutionary process, plants have gradually developed roots that are adapted to environmental changes. Based on morphological characteristics, plant roots can be primarily classified into taproot systems and fibrous roots. Among these, taproot systems are commonly observed in most dicotyledonous plants, such as grapes. A taproot system consists of a primary root (PR), lateral roots (LRs), and adventitious roots (ARs) (Figure 1A). The primary root is well-developed and robust; lateral roots exhibit distinct branching hierarchies; and adventitious roots serve to support the above-ground parts of the plant and absorb nutrients from the soil surface layer. The primary root, also referred to as the initial root, is mainly formed through the continuous division and growth of the root apical meristem. Structurally, from the outer to the inner layer, it includes the epidermis, cortex, endodermis, pericycle, and vascular tissue that consists of xylem and phloem [20] (Figure 1C). The pericycle cells are composed of two types: one type is associated with the phloem and represents differentiated cells; the other type corresponds to the xylem and consists of meristematic cells. From the morphological base to the apex, the primary root is structured as the mature zone, elongation zone, meristematic zone, and root cap (Figure 1B). Lateral roots originate from the pericycle cells corresponding to the xylem. Their development involves four key stages: periclinal division and anticlinal division of pericycle cells, formation of lateral root primordia, and emergence of lateral roots (when lateral root primordia penetrate the epidermis). Adventitious roots initiate from pericycle cells, parenchyma cells, or cambium cells. Their development can be divided into three main phases: the induction phase, the initiation phase, and the expression phase [21].

**Figure 2:**
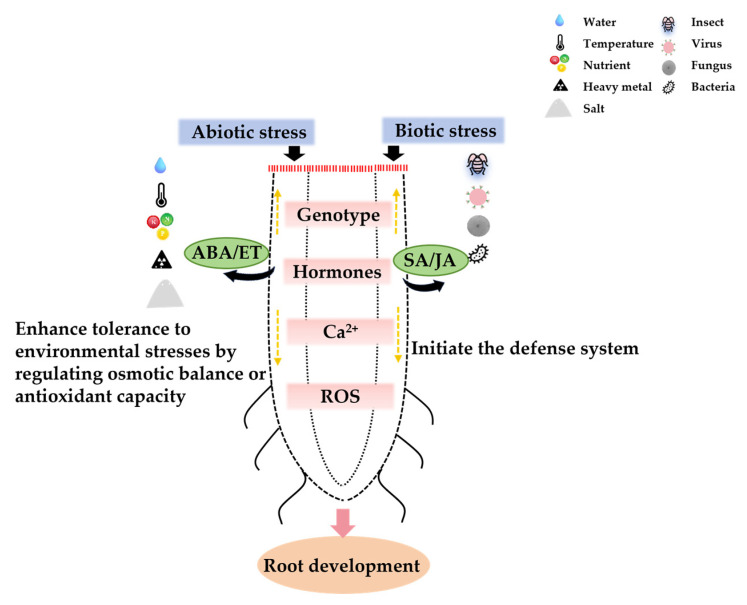
Factors affecting root development. This diagram illustrates the conserved signal transduction cascades in fruit crop roots under abiotic (water deficit, nutrient fluctuation, salinity, etc.) and biotic (insect, virus, fungal, and bacterial pathogens) stresses. Abiotic stress triggers the ABA/ET (abscisic acid/ethylene) signaling pathway, which enhances tolerance by regulating osmotic balance and antioxidant capacity, thereby reducing oxidative damage. Biotic stress activates the SA/JA (salicylic acid/jasmonic acid) defense pathway, initiating the plant immune response. Both stress types converge on shared downstream signaling components, including genotype-dependent hormonal regulation, Ca^2+^, and ROS production, which collectively modulate root development and stress adaptation.

As a crucial organ for plants to absorb water and nutrients, the root plays a vital role in the growth, development, and stress adaptation of fruit trees. Firstly, a well-developed and healthy root can significantly enhance the water and nutrient absorption capacity of grape plants. Secondly, the root anchors the plant in a specific location and is responsible for the transportation, synthesis, and storage of nutrients. In fact, the growth status of the root often determines the growth and development of above-ground organs and the yield of the tree. The growth and development of the root are regulated by multiple internal and external factors (Figure 2). Internal factors mainly include plant genotypes, hormones and various signaling molecules, while external factors encompass various nutrient elements, water availability, light conditions, and temperature, which modulate the development of lateral roots and adventitious roots mainly by influencing internal factors [22,23,24]. For instance, auxin participates in all stages of lateral root development in fruit trees, or is involved in regulating the formation of adventitious roots on the hypocotyl of various plants through its polar transport [25], or induces the initiation of adventitious roots [26].

The root signaling module in fruit trees enables precise responses to abiotic stresses through a hierarchical regulatory mechanism of ‘perception–transduction–response’. Initially, receptor-like protein kinases (RLKs) on the surface of root cells act as the front-line sensors. These can recognize environmental signals such as drought and salinity. For example, the receptor kinase FERONIA in root epidermal cells can detect cell wall damage signals and regulate the root’s adaptive response to drought [27].

## 3. Perception of Stressors

**Figure 3:**
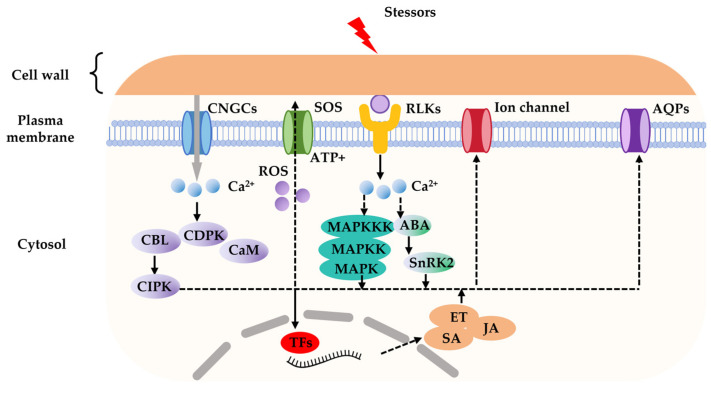
Schematic diagram of abiotic atress aignal transduction in fruit crops’ roots. This diagram depicts the core signaling events triggered by environmental stressors at the root cell, leading to transcriptional and physiological responses: Stress perception: stress signals are detected by plasma membrane-localized receptors (e.g., RLKs) and sensors, including CNGCs, SOS Na^+^/H^+^ antiporters, and AQPs. Early signal generation: stress perception induces the rapid influx of Ca^2+^ into the cytosol and the production of ROS. These secondary messengers activate calcium-sensing proteins such as CBL, CIPK, CDPK, and CaM. Signal amplification: the signal is relayed through phosphorylation cascades, including the MAPK (mitogen-activated protein kinase) module (MAPKKK → MAPKK → MAPK) and SnRK2 (SNF1-related protein kinase 2) kinases, which also integrate ABA signaling. Transcriptional and hormonal regulation: activated signaling components translocate to the nucleus to modulate TFs, or induce the synthesis of defense hormones such as SA, JA, and ET, thereby initiating stress-responsive gene expression and physiological adaptations. Physiological adjustments: the signaling cascade also directly regulates plasma membrane ion channels and aquaporins to maintain cellular ion and water homeostasis under stress.

Plant cell membrane protein receptors are generally classified into four major categories: RLKs initiate phosphorylation cascades by perceiving environmental signals; ion channels respond to fluctuations in soil ion concentrations and osmotic pressure, mediating the regulation of ion fluxes such as potassium and calcium; non-selective cation channels (NSCCs) maintain ion homeostasis through ion transport; aquaporins (AQPs) participate in water transport and signaling molecule transduction. These transmembrane proteins function synergistically to form the molecular basis for fruit tree root adaptation to abiotic stress, which is of great significance for elucidating the regulatory mechanisms underlying stress tolerance (Figure 3).

### 3.1. RLKs

RLKs are critical signal recognition proteins localized on the plant cell membrane. Structurally, RLKs consist of extracellular domains, transmembrane domains, and intracellular kinase domains. The extracellular domains specifically recognize a wide range of environmental signals, including peptide hormones, cell wall damage signals, and pathogen-associated molecular patterns. Upon signal recognition, RLKs undergo autophosphorylation or form heterodimers with other RLKs, thereby activating their intracellular kinase domains. This activation further recruits downstream signaling molecules and initiates signal transduction cascades, which regulate root growth, development, and stress responses in fruit tree species such as apple [28] and strawberry [29]. For instance, the cysteine-rich RLK (CRK) family perceives abiotic stress signals (e.g., drought and salinity) via its extracellular domains. The structural conservation and functional diversity of CRKs—for example, CRK28 and CRK36 participate in ROS production and stomatal closure—provide a molecular basis for root stress perception [30].

### 3.2. Ion Channels

Ion channels are highly sensitive to fluctuations in soil ion concentration and osmotic pressure. When root cells perceive osmotic stress, water loss induces osmotic pressure changes that activate specific ion channels and receptors, converting external stimuli into intracellular ion fluxes to trigger downstream signaling pathways. In fruit trees, ion channels are diverse and functionally specialized, mainly including selective ion channels and NSCCs, which play essential roles in growth, development, and environmental stress adaptation.

#### 3.2.1. Selective Ion Channels

Selective ion channels mediate passive transport of specific ions and exhibit strict ion specificity, ensuring precise regulation of intracellular ion homeostasis.

Potassium (K^+^) channels: Predominantly members of the Shaker family, and categorized into inward- and outward-rectifying types. For example, VvK1.1 in grape is an inward-rectifying K^+^ channel that enhances K^+^ uptake under salt stress, maintaining cellular K^+^/Na^+^ balance. Outward-rectifying K^+^ channels mediate K^+^ efflux upon membrane depolarization, regulating cell turgor pressure and ion excretion [31].

Chloride (Cl^−^) channels: Including voltage-gated subtypes, they participate in plant defense responses through anion (Cl^−^, NO_3_^−^) transport. Key functions include regulating stomatal movement, stabilizing membrane potential, supporting photosynthesis, maintaining pH balance, and mediating electrical signaling [32,33,34]. In grapes, Cl^−^ exclusion mechanisms are constitutively active and variety-specific, rather than stress-inducible [35].

#### 3.2.2. NSCCs

NSCCs mediate passive transport of multiple cations without strict specificity, primarily functioning in rapid transmission of stress signals. The core member in fruit trees is the cyclic nucleotide-gated channels (CNGCs) family. Unlike animals, plants lack voltage-gated Ca^2+^ channels, but CNGCs serve as critical Ca^2+^-permeable non-selective channels central to ABA-induced stomatal closure [36,37]. For example, MdCNGC2 in apple roots mediates Ca^2+^ influx under salt stress, activating downstream ABA signaling pathways to enhance stress tolerance [38].

### 3.3. Selective Cation Transporters

Selective cation transporters rely on active transport driven by energy or ion gradients, specifically transporting 1–2 types of cations to maintain ion homeostasis under normal growth and stress conditions. In fruit trees, key members include four major families:

1. Plasma membrane-localized salt overly sensitive 1 (SOS1) Na^+^/H^+^ antiporters: Driven by the proton gradient generated by H^+^-ATPases, SOS1 acts as the first line of defense against salt stress in apple [39], grape [40], and peach [41]. As a Na^+^/H^+^ antiporter, SOS1 senses elevated intracellular Na^+^ levels via auto-inhibitory domains. Under salt stress, the SOS2–SOS3 complex phosphorylates SOS1, relieving auto-inhibition and activating Na^+^ efflux to maintain ion balance [42]. Emerging evidence suggests SOS proteins also regulate root development beyond Na^+^ homeostasis: SOS3 modulates lateral root plasticity by regulating auxin gradients under mild salt stress, and SOS proteins participate in cytoskeletal dynamics under stress [43];
2. Vacuolar Na^+^/H^+^ exchanger (NHX)-type Na^+^/H^+^ antiporters: These transporters compartmentalize cytoplasmic Na^+^ into vacuoles, reducing ion toxicity and maintaining cellular osmotic balance. A typical example is FaNHX1 in strawberry [44];
3. High-affinity K^+^ transporter (HKT) family transporters: They selectively regulate Na^+^ and K^+^ transport, controlling xylem Na^+^ unloading and shoot K^+^/Na^+^ balance. For instance, VcHKT1;1 in blueberry plays a key role in this process [45];
4. Cation/Ca^2+^ exchanger (CCX) family: These transporters mediate Na^+^ extrusion via Na^+^/Ca^2+^ exchange and regulate intracellular Ca^2+^ homeostasis. For example, MdCCX2 in apple is critical for responses to salt and other stresses [46].

### 3.4. Other Transmembrane Proteins

The aforementioned ion channels function coordinately to regulate ion homeostasis and physiological processes in fruit trees. In addition, other transmembrane signaling proteins, such as AQPs, also contribute to root responses to adverse environments. AQPs not only facilitate water transport but also act as second messengers by transporting signaling molecules (e.g., H_2_O_2_, NO, CO_2_) in grape [47], apple [48], and citrus [49,50]. Alternatively, they indirectly activate mechanosensitive signals by regulating cell turgor pressure [51]. AQPs can also serve as direct targets of ABA signaling or TFs, participating in the reprogramming of gene expression [52].

## 4. Transmission and Transduction

The MITOGEN-ACTIVATED PROTEIN KINASE (MAPK) cascade represents a central signaling pathway in plants for responding to environmental stress. Under root stress conditions, signals are transmitted through a three-tiered phosphorylation cascade involving MAPKKK → MAPKK → MAPK. Once activated, MAPK translocates into the nucleus and phosphorylates downstream TFs or enzymes, thereby regulating gene expression and physiological adaptations [53,54,55]. For instance, RNA-Seq analysis of the apple rootstock *Malus hupehensis* under NaCl and KCl stress revealed that multiple MAPK signaling-related genes are involved in maintaining Na^+^ and K^+^ homeostasis in the root system [56]. Overexpression of *VvMKK2* significantly enhances tolerance to both salt and drought stress, whereas overexpression of *VvMKK4* only improves salt stress tolerance [57]. Proteomic studies have shown that the phosphorylation levels of MAPK-related proteins, such as MPK4, are markedly elevated in roots under salt stress [58]. Moreover, emerging evidence indicates that the MAPK signaling pathway plays a pivotal role in plant responses to heavy metals by inducing intracellular antioxidant mechanisms [59].

As a critical transitional phase in the root stress response, signal transduction bridges the gap between initial signal detection and functional execution, and can be categorized into two major pathways: hormone-mediated signal transduction, and second messenger systems.

### 4.1. Hormones

ABA serves as a central regulator in plant responses to abiotic stress [60]. Under drought conditions, two major signaling pathways—ABA-dependent and ABA-independent—are activated. In the ABA-dependent pathway, the coordination between stomatal closure and root water uptake is essential for maintaining water balance. Upon drought perception, the ABC transporter ABCG25 facilitates the transport of root-synthesized ABA and its glucose ester (ABA-GE) to aerial tissues via xylem and phloem, thereby regulating stomatal movement and reducing transpirational water loss [41]. Simultaneously, ABA activates SnRK2 protein kinases, which phosphorylate AREB/ABF TFs to regulate the expression of downstream drought-responsive genes [61,62]. In contrast, the ABA-independent pathway enables the root to enhance water absorption through morphological adjustments and the synthesis of osmoprotective substances.

ET, as a gaseous hormone, plays an indispensable role in root responses to abiotic stress. It operates through the classical ethylene response 1-constitutive triple response 1-ethylene insensitive 2/ethylene response factor (ETR1-CTR1-EIP2/ERF) signaling pathway [63]. Under stress conditions such as salinity, drought, and cold, root cells perceive environmental signals and activate key enzymes for ET biosynthesis—ACC synthase (ACS) and ACC oxidase (ACO)—leading to elevated ET levels. ET suppresses CTR1 kinase activity, thereby relieving the phosphorylation inhibition of EIN2 and allowing its translocation into the nucleus to activate EIN3/EIL1 TFs. These factors regulate the expression of downstream ERFs, which act as central regulators by binding to cis-elements such as GCC-box or DRE, thereby coordinating the expression of stress-related genes including ion transporters, antioxidant enzymes, and osmoprotective substance synthases. This ultimately enhances root adaptability under adverse conditions [24].

Auxin maintains root polarity under normal growth conditions and reprograms root architecture under stress. Under drought stress, auxin levels decrease in the root apical meristem, inhibiting primary root elongation, while auxin accumulates in lateral root primordia, promoting lateral root development and expanding the root absorption surface [64,65,66,67,68]. During this process, the expression and localization of auxin biosynthesis and transport proteins (e.g., YUC and PIN) are regulated by multiple environmental and hormonal signals, thereby shaping auxin distribution [69,70]. For instance, 12 *VvPIN* genes have been identified in grapevine, with their promoters containing cis-regulatory elements responsive to light, abiotic stress, and hormones. Most of these genes are significantly induced by hormones such as naphthaleneacetic acid (NAA), gibberellic acid 3 (GA3), and ABA, as well as stresses including NaCl, cold, and PEG [71]. Heterologous overexpression of *VaIAA3* enhanced cold tolerance [72]. Under salt stress, overexpression of *VvIAA18* significantly increases root auxin levels in grapevine, activates antioxidant genes (e.g., *VvSOD*, *VvCAT1*), and osmotic adjustment genes, thereby improving salt tolerance [73].

Notably, multiple hormone signaling pathways are intricately interconnected and function synergistically in root stress responses, forming a complex regulatory network to cope with diverse adverse conditions. Under salt stress, ABA promotes ET biosynthesis, while ET enhances ABA signaling, forming a positive feedback loop that induces the expression of ion transporter genes, thereby promoting Na^+^ efflux and K^+^ uptake and maintaining cellular ion homeostasis. For example, under drought stress, SnRK2.4 kinase in citrus (*Poncirus trifoliata*) is activated by ABA and phosphorylates the ABF2 transcription factor at Ser93, enhancing its binding to the ABRE element in the *ADC* gene promoter, thereby activating *ADC* expression and promoting putrescine synthesis to enhance drought tolerance [74]. ABA and auxin also interact under drought stress: ABA inhibits auxin transporters, altering auxin distribution in roots and thereby regulating root growth direction and morphology. Simultaneously, they interact through key signaling components to co-regulate the expression of downstream stress-responsive genes. The interaction between ABA and indole-3-acetic acid (IAA) during chilling stress may be modulated by ET signaling, suggesting that these hormones collectively form an integrated hormonal signaling network underlying cold stress responses in grapevine [75]. Transgenic Arabidopsis overexpressing *VaIAA3* exhibited enhanced cold tolerance through the regulation of auxin, ABA, and ET signaling pathways [72].

### 4.2. Second Messengers

#### 4.2.1. Ca^2+^ (Ca^2+^ Sensors, CaM/CaMKs)

As a versatile second messenger, Ca^2+^ plays essential roles in nearly all abiotic stress responses in plant roots. For any given abiotic stress, Ca^2+^ is not only closely associated with stress perception but also orchestrates the subsequent signal transduction cascades through multiple functional mechanisms. When the root system perceives stresses such as low temperature and drought, changes in cell membrane permeability lead to the opening of Ca^2+^ channels and a rapid increase in intracellular Ca^2+^ concentration, generating a specific Ca^2+^ signal fingerprint [76,77]. Processes regulated by Ca^2+^ sensors contribute to the homeostasis of macronutrients such as nitrogen (N), magnesium (Mg^2+^), and micronutrient iron (Fe), thereby preventing nutrient deficiency in the root systems of fruit trees [78]. Under heavy metal stress, plants exhibit altered Ca^2+^ channel function and increased Ca^2+^ influx into cells. Subsequently, calcium activates calmodulin, which is a key regulatory protein that controls the uptake, transport, and metabolism of heavy metals [79].

These signals are recognized by Ca^2+^ sensors such as calmodulin (CaM), calcineurin B-like proteins (CBLs, including CBL1–CBL6, CBL8–CBL10), and SOS3-like calcium binding protein3 (SCaBP3). Upon Ca^2+^ binding, sensors such as CBL7, SCaBP3, or CaM undergo conformational changes that activate downstream CBL- CIPKs or calmodulin-dependent protein kinases (CaMKs). These kinases regulate root development and enhance abiotic stress tolerance by modulating ion channels (e.g., Na^+^/H^+^ antiporters), transporters (e.g., VvPMA10), and TFs (e.g., VvMYC2) [80,81]. Additionally, exposure to apple replant disease (ARD) soil extract affects Ca^2+^ currents in the rhizoplane zones of different apple rootstocks (12-2, T337, M26) with varying degrees depending on exposure time, and such Ca^2+^ current changes are associated with the rootstocks’ resistance to ARD [82].

For example, under low-temperature stress, Ca^2+^ concentration in root cells rises, activating the Ca^2+^-CBL-CIPK signaling pathway. This pathway maintains cellular ion homeostasis by regulating K^+^ channel activity, thereby enhancing plant cold tolerance [83]. The Ca^2+^-CBL1 complex activates VvCIPK23, whose NAF/FISL motif is de-repressed through conformational changes under cold conditions. This activation enhances the stability of the transcription factor VaMYB4a via phosphorylation at Ser133/Ser182 residues, thereby inducing the expression of C-REPEAT BINDING FACTOR (CBF) and improving grape cold resistance [84]. Under cold stress, *VvCBL1* and *VvCIPK23* are significantly upregulated in roots, and the kinase activity of VvCIPK23 is regulated by the Ca^2+^-CBL1 complex. Moreover, CPK21 and CPK23, as calcium-dependent protein kinases, induce Ca^2+^ signaling and transcriptional reprogramming under aluminum stress, promoting the expression of Al-resistance genes mediated by the transcription factor STOP1 and regulating root malate efflux to limit aluminum accumulation at root tips [85,86].

Salt stress rapidly elevates intracellular Ca^2+^ levels. SOS3, containing EF-hand domains, specifically binds Ca^2+^. Upon Ca^2+^ binding, SOS3 undergoes a conformational change, exposing interaction sites for SOS2, which recruits and activates SOS2 to initiate downstream signaling that activates ion transporters and maintains intracellular ion homeostasis [87]. In fruit trees, the SOS pathway (SOS3-SOS2-dependent) and the CBL-CIPK pathway may cooperatively regulate ion transport through shared Ca^2+^ signaling. Specifically, SOS3-mediated activation of SOS2 preferentially responds to early Ca^2+^ transients under high salinity, while the CBL-CIPK pathway contributes to fine-tuning ion homeostasis under prolonged salt stress. For instance, the grapevine CBL1-CIPK23 module maintains K^+^ uptake by phosphorylating the K^+^ channel VvK1.1, whereas the SOS pathway mediates Na^+^ extrusion via SOS1, collectively preserving K^+^/Na^+^ balance [88].

#### 4.2.2. ROS and NO Signals

ROS and NO are not only byproducts of stress responses but also crucial signaling molecules involved in plant adaptation to abiotic stress [89,90]. Under abiotic stress, ROS levels—including superoxide anions (O_2_^−^) and hydrogen peroxide (H_2_O_2_)—increase in root cells. At early stages, low concentrations of ROS act as signaling molecules that activate antioxidant enzyme systems such as superoxide dismutase (SOD) and peroxidase (POD), thereby scavenging excess ROS and maintaining cellular redox homeostasis [91,92]. For example, ROS can initiate stress responses in apple roots under iron deficiency [93], or interact with MxRop1 and MxRBOHD1 to regulate ROS-mediated adaptive responses in *Malus xiaojinensis* [94]. In addition, ROS interacts with hormones and other signaling molecules to regulate the expression of resistance genes and modulate protein activity, thereby enhancing plant stress tolerance [95]; for example, ROS contributes to drought and salt tolerance in apple [96,97], waterlogging in strawberry [98], and salt resistance in grapevine [99].

Furthermore, NO can act as a signaling molecule by modulating oxidative stress, antioxidant defense, metal transport, and ion homeostasis. NO application alleviates iron deficiency symptoms in *Malus micromalus* [100], enhances osmotic stress tolerance in banana roots [101], improves cold resistance in peach [102], and mitigates iron deficiency or salinity stress in strawberry [103]. Moreover, NO interacts with various signaling molecules—including gasotransmitters, hormones, ions, and polyamines—to form complex regulatory networks [104,105]. For example, the synergistic interaction between NO and H_2_O_2_ initiates mechanisms such as Na^+^ exclusion, vacuolar sequestration, and Na^+^ removal from xylem sap, thereby limiting Na^+^ accumulation, promoting Na^+^ homeostasis, and enhancing salt tolerance in strawberries and grapevines [106,107]. Other gaseous signaling molecules, such as hydrogen sulfide (H_2_S), also regulate root responses in fruit trees. In peach roots, H_2_S promotes lateral root formation through sulfhydrylation of SnRK1α kinase, thereby improving adaptation to waterlogging stress [108,109,110].

Moreover, these signaling pathways often crosstalk to regulate root growth and development, with obvious concentration effects and species specificity (Table 1). Specifically, the aforementioned signals, including hormone, Ca^2+^, and ROS/NO pathways, form a rigorous synergistic regulatory network via core nodes. While activating SnRK2 to initiate downstream responses, ABA induces an increase in intracellular Ca^2+^ concentration, which enhances ion homeostasis regulation through the CBL-CIPK module; conversely, ROS can phosphorylate SnRK2 in a feedback manner to amplify the ABA signal [62,84,95]. The ET and Ca^2+^ pathways interact through the VvERF1B-VvMYC2-VvPMA10 pathway [81] and mediate the cross-regulation between ABA and auxin [75]. The expression of auxin PIN proteins is co-regulated by Ca^2+^ and ROS, thereby reshaping root architecture [71,111]. NO and H_2_O_2_ synergistically maintain Na^+^ homeostasis and transmit signals via Ca^2+^ sensors, while Ca^2+^ can activate the MAPK pathway to regulate ROS synthesis, forming a multi-pathway interaction loop [86,106,112].

Collectively, Ca^2+^, ROS, and NO as second messengers not only mediate independent signal transduction cascades but also interact closely with hormone signaling pathways, jointly regulating root adaptation to abiotic stress.

## 5. Physiological Responses

The physiological and biochemical mechanisms underlying the response of fruit crops roots to abiotic stress involve multi-dimensional regulatory strategies. Fruit crops adapt to adverse environmental conditions through three major mechanisms: root morphological remodeling [113]; regulation of osmotic adjustment substance synthesis to maintain cellular osmotic balance [114]; and modulation of the antioxidant enzyme system to preserve cellular redox homeostasis and enhance root stress tolerance (Figure 3) [115].

Firstly, under abiotic stress, fruit crops roots undergo significant morphological adaptations [116]. For example, nitrogen and/or phosphorus deficiency suppresses shoot growth, increases root allocation of total N and P, enhances root tip number, length, volume, and surface area, improves the root-to-shoot ratio, and alters the biosynthesis of cell wall components such as cellulose, hemicellulose, lignin, and pectin in *Malus domestica* borkh [117]. Similarly, *Pyrus betulifolia* bunge rapidly reprograms its transcriptome under potassium deprivation to promote root growth and enhance potassium uptake [118]. Under drought conditions, fruit crops exhibit accelerated primary root growth and deeper root penetration, while lateral root development is suppressed, resulting in reduced root length and surface area. These morphological changes minimize water loss and enable roots to access deeper soil layers for water acquisition [119]. Citrus species modify root morphology and biological traits as a nutrient acquisition strategy to maximize micronutrient uptake and sustain growth under stress [120]. Salt-alkali stress also inhibits root growth, leading to reductions in root length, surface area, and volume, along with decreased root vitality, which compromises nutrient and water uptake in grapevines and apple trees [121,122]. However, within a certain salinity range, the salt-tolerant apple rootstock ‘9-1-6’ maintains low salt sensitivity and a high root-to-shoot ratio by increasing root biomass allocation and limiting Na^+^ translocation to the shoot, thereby enhancing stress tolerance [123]. Direct root-zone irrigation, a novel subsurface irrigation technique initially tested in vineyards to conserve water and ensure grape production in arid regions with unstable climates, promotes deeper root development for improved water uptake and alleviates drought stress [124]. Under drought, grapevine rootstocks with higher root length density (RLD) and a greater proportion of fine roots maintain Ψstem more effectively during severe drought. Additionally, smaller xylem vessel diameters and reduced xylem area relative to root cross-sectional area correlate with enhanced water transport efficiency and faster recovery post-drought [125]. Increased root hair length also contributes to improved drought tolerance in grapevines [126]. In pecans, prolonged drought reduces root biomass, average diameter, root tissue density, and cortex thickness, while increasing specific root length, stele diameter, and conduit density [127].

Secondly, as stress intensity increases, plant roots maintain cellular osmotic and ionic homeostasis through the synthesis and accumulation of osmolytes, along with the regulation of ion absorption and transport mechanisms, thereby ensuring normal physiological metabolism [128,129]. Under drought and salinity stress, fruit crops roots induce the expression of genes involved in the biosynthesis of osmotic regulators such as proline and betaine, which promote proline accumulation, lower cellular osmotic potential, enhance water retention capacity, and sustain normal physiological functions [130,131,132]. Salt-tolerant fruit crops regulate ion uptake and transport to maintain ionic balance by limiting Na^+^ absorption, enhancing K^+^ uptake and translocation, and sustaining a higher intracellular K^+^/Na^+^ ratio, as observed in blueberries [42], jujubes [133], and grapes [134].

Additionally, abiotic stress often triggers the overproduction of ROS in roots, leading to oxidative damage. To counteract this, ROS accumulation activates signaling modules that induce the antioxidant enzyme system. Activated MAPK cascades and other signaling pathways regulate the expression of antioxidant enzyme genes such as SOD, POD, and CAT, thereby enhancing enzymatic activity and efficiently scavenging excess ROS to mitigate oxidative damage [135]. For example, the flood-tolerant wax myrtle (*Morella cerifera*) rootstock accumulates higher levels of antioxidant enzyme activity and differentially expressed genes—such as those enriched in the MAPK, glycolysis/gluconeogenesis pathways—under root zone hypoxia [136]. Nitric oxide (NO) and ROS work synergistically to regulate the antioxidant system and jointly maintain cellular redox balance. In strawberries, FaWRKY40 binds to the promoters of *FaRbohD*, *FaNHX1*, and *FaSOS1*, participating in the maintenance of root Na^+^ homeostasis and enhancement of salt tolerance through a transcriptional regulatory mechanism involving hydrogen peroxide (H_2_O_2_) and nitric oxide (NO) signaling cascades [109].

## 6. Regulatory Factors

Throughout the entire process of signal perception, transduction, and response, TFs and epigenetic modifications play central regulatory roles, functioning as global modulators that cross-connect multiple signaling pathways.

### 6.1. TFs

TFs such as MYB, WRKY, bHLH, NAC, and AP2/ERF act as key nodes in integrating upstream signals and regulating downstream stress-responsive genes. Unlike pathway-specific components, these TFs often mediate crosstalk between multiple stress responses, and many of them are phosphorylated and activated by upstream MAPK cascades or regulated by ROS signaling to amplify signal transduction across different stress pathways (Table 2).

MYB TFs are extensively involved in the tolerance of fruit crops to various abiotic stresses by regulating hormone signaling, antioxidant systems, and metabolic pathways. VvMYB108A from grapevine enhances salt tolerance by modulating the ET signaling pathway [137]; MdMYB308L from apple interacts with MdbHLH33 to positively regulate cold tolerance, and its protein stability is controlled by MdMIEL1-mediated degradation [138]; heterologous expression of apple *MbMYB4*, *MbMYB108*, and grapevine *VhMYB2/15/60*, *VaMYB14* significantly improves the resistance of transgenic plants to cold, drought, or salt stress [139,140,141,142,143]; and in the crosstalk between drought and cold stress responses mediated by apple, MhZAT10, MhMYB88 and MhMYB124 act as key interacting factors [144,145].

WRKY TFs exert stress-resistant functions by regulating ion balance, ROS scavenging, and the expression of stress-responsive genes. In apple, MPK3/MPK6 phosphorylate MdWRKY17 to activate *MdASMT7* expression, enhancing tolerance to drought, salt, and oxidative stress through ROS scavenging and proline regulation [146]; WRKY53 regulates MdGORK1 to promote K^+^ efflux, maintaining ion homeostasis under KCl stress [147]; MdWRKY115 directly binds to the *MdRD22* promoter to improve drought and osmotic stress resistance [148]; heterologous expression of apple MbWRKY50 in tomato enhances cold and drought tolerance by binding to CBF/DREB elements or participating in ABA synthesis [149]; kiwifruit AcWRKY117 and AcWRKY29 transcriptionally activate the *AcPDC2* gene to respond to waterlogging stress [150]; and grapevine VaERF092 binds to the GCC-box in the *VaWRKY33* promoter to improve cold tolerance [151]. Additionally, ROS signaling activates the MAPK cascade, which regulates WRKY TFs to participate in ion absorption, transport, and detoxification processes, maintaining root physiological homeostasis [152].

bHLH TFs mediate stress responses by regulating metabolic pathways, ROS balance, and enzyme activity. Apple bHLH130 regulates flavonoid and lignin biosynthesis pathways to improve nitrogen absorption efficiency of rootstocks under low-nitrogen stress [153]; MdbHLH160 promotes the accumulation and activity of nuclear MdSOD1, inhibiting excessive ROS accumulation, and its stability under drought/ABA conditions is maintained through ABA-inhibited MdBT2-mediated ubiquitination/degradation [154]; grapevine VvbHLH036 regulates threonine synthase biosynthesis to enhance root cold tolerance [155]; and in iron-efficient apple rootstocks, the MxMPK6-2-MxbHLH104 pathway is activated to improve iron deficiency tolerance [111].

NAC TFs participate in stress resistance by regulating ion transport and the expression of stress-responsive genes. Under salt stress, they can modulate the expression of ion transporter genes to maintain ion homeostasis; for example, PbeNAC1 from *Pyrus betulifolia* interacts with PbeDREB1 and PbeDREB2A to enhance the expression of stress-associated genes, improving stress tolerance [156]. Additionally, signaling molecules such as ROS and Ca^2+^ can activate NAC TFs, which in turn regulate the expression of stress-responsive genes [157].

The AREB/ABF subfamily of the bZIP family is activated by ABA signals and binds to ABRE elements to regulate drought-responsive genes; apple MdDREB2A is functionally associated with bZIP-mediated stress responses, positively regulating nitrogen utilization by binding to the DRE cis-element in the MdNIR1 promoter and coordinating carbon–nitrogen redistribution under drought [158]; and ROS-MAPK cascade signaling can also regulate bZIP TFs to participate in the maintenance of root physiological homeostasis [152].

**Table 2:** Regulatory factors of aboitic stresses in fruit tree root.

Stressors		Regulatory Factor	Species	References
Root restriction	*CHS*	lncRNA/circRNA	Grapevine	[159]
	ROS	miRNAs	Apple	[160]
Nutrient limitation	ABA	MdbHLH130/160; MdDREB2A	Apple	[153,158]
	*MhNRT2.4*	miR164-MhNAC1		[161]
	*Mdα-GP2*	MdbZIP44		[162]
	*FaVPT1*	FaSCL8	Strawberry	[163]
Water stress	ROS	MbMYB108	Apple	[139]
	ABA-independent	MhZAT10		[145]
	ABA	MdWRKY29/50; MdbHLH160		[149,154,164]
	Cytokinins	MdbZIP80		[165]
	-	VhMYB2; VaMYB15	Grapevine	[141,143]
	GA	VyMYB24		[166]
	-	VvNAC17		[167]
	-	AcWRKY117/29	Kiwifruit	[150]
	-	FvICE1	Strawberry	[168]
	Putrescine	FcWRKY70	Citrus	[169]
	ABA	PtrbHLH66		[170]
Salt stress	H+-ATPase	VvERF1B	Grapevine	[81]
	ET	VvMYB108A		[137]
	Osmolytes	VvWRKY2		[171]
	Auxin	MdSIMYB1	Apple	[172]
	-	MbWRKY33		[173]
	-	MdbZIP		[174]
	ABA	PpcMYB20	Pear	[175]
	H+-ATPase	PbbHLH62		[176]
	-	PacMYBA	Sweet cherry	[177]
	-	CsMYB30	Citrus	[178]
Extrem-temperature stress	ABA-independent	MhDREB2A/MhZAT10 MbWRKY50	Apple	[145]
	ABA			[149]
	ET, ABA, and methyl jasmonate	VaMYB14/44	Grapevine	[179]
	-	VaWRKY72		[180]
	-	PubHLH1	Pear	[181]
	-	FvICE1	Strawberry	[168]
	Polyamine	PtrICE1	Citrus	[182]
Heavy metal	-	lncRNA/circRNA	Citrus	[183]
	-	miR156	Grapevine	[184]
	H^+^ transport and ion gradients	MdSTOP1-MdNAC2	Apple	[185]
	-	MdWRKY17		[186]
	ABA	WRKY40/ERF98/NAC2	Strawberry	[187]

ERF TFs are mainly involved in stress tolerance by regulating substance metabolism. For example, 5 salt-inducible AP2/ERF genes were identified from a total of 131 in sweet orange, showing significant responses to salt stress [188]. Overexpression of *HuERF1* enhanced salt tolerance, reduced ROS accumulation, and improved antioxidant enzyme activities in pitaya [189]. In citrus tetraploid rootstocks, genome doubling enhances the ET signaling pathway, activating ROS-scavenging genes and ion transporters, thereby reducing Na^+^ accumulation and membrane damage in roots [58,190]. In grapevine, exogenous ET strongly induces *VvERF1B* expression, which enhances NaHCO_3_ tolerance by increasing lipid membrane proton pump activity and promoting H^+^ and oxalate secretion [81]. The grapevine VaEIN3.1-VaERF057 module positively regulates cold tolerance by modulating soluble sugar content [191]. Heterologous expression of apple *MhZAT10* can enhance the drought and cold tolerance of sensitive rootstocks, with MhDREB2A-MhWRKY31 serving as key downstream interacting factors [145].

### 6.2. Epigenetic Modifications

Epigenetic regulation includes non-coding RNAs, methylation, and protein modification, which regulates the expression of genes related to stress-responsive signaling pathways through ROS homeostasis, various hormone signals, and other mechanisms, thereby mediating the adaptability of plants to abiotic stresses [192,193,194,195].

For instance, under root-restriction conditions, *lncRNAs* and *circRNAs* act as competing endogenous RNAs (ceRNAs) to regulate grape root development-related pathways, indirectly modulating root ROS-scavenging capacity and auxin signal transduction, and thus optimizing plant adaptability to stress [196]. The miR156ab-SPL13 module positively regulates auxin-related genes, modulates auxin metabolism and antioxidant enzyme activity, and promotes lateral root formation, thereby enhancing drought resistance in apple [197,198,199]. Under low-temperature stress, miR528 regulates the expression of its target gene *MaPPO* (polyphenol oxidase), inducing ROS accumulation to activate defense genes and enhance banana chilling tolerance [200]. Under copper toxicity stress, the ceRNA regulatory network mediates pathway coordination by enabling non-coding RNAs to regulate downstream genes, thereby alleviating copper-induced ROS burst and maintaining ROS signaling pathway homeostasis in citrus [183]. Meanwhile, miR156 can enhance apple salt tolerance by regulating the salt stress response pathway and its downstream target genes, and this regulatory process is closely associated with the ROS homeostasis and ion balance pathways [159].

At the transposon methylation level, methylation of the miniature inverted-repeat transposable element in the promoter of apple *MdRFNR1* activates the gene, maintaining ROS homeostasis and enhancing drought response [160]. In apple, MdBPC2 forms a complex with the polycomb protein LHP1, and specifically represses the expression of key auxin synthesis genes *MdYUC2a* and *MdYUC6b* via histone H3K27 methylation. This repression attenuates auxin signal transduction, thereby affecting plant tolerance to stress [201].

Protein modifications include SUMOylation and acetylation, which share similar regulatory mechanisms. For example, MdDREB2A in apple is a core regulator of the drought response pathway. Both the increase and decrease in the SUMOylation level of MdDREB2A can enhance its functional activity, promote the expression of downstream drought-resistant genes (including ROS-scavenging related genes), improve plant drought tolerance, and achieve bidirectional and precise regulation of the drought response pathway [202]. Apple histone deacetylase 6 (MdHDA6) catalyzes the deacetylation of histones associated with drought-responsive genes, which represses the transcription of downstream drought-resistant genes (including genes involved in ROS homeostasis regulation) and weakens the drought stress response of plants [203].

## 7. Biotechnological Applications

### 7.1. Genetic Engineering

Significant progress has been achieved in the application of genetic engineering to fruit crop roots. Modern genetic engineering encompasses Agrobacterium- or plasmid-mediated gene transformation, as well as genome editing technologies that enable precise modification of target DNA sequences (e.g., insertion, deletion, substitution), thereby facilitating the improvement of desired traits [204]. Among these, clustered regularly interspaced short palindromic repeats (CRISPR)/CRISPR-associated protein (Cas) systems, zinc finger nucleases (ZFN) and transcription activator-like effector nucleases (TALEN) allow precise gene editing, which can directly target core genes of root signal modules to verify their functions, clarify unclear regulatory mechanisms, and accelerate the breeding of stress-tolerant varieties [205], particularly in species such as apple, grape, and citrus [206,207]. For example, in response to drought stress, researchers targeted the *glutathione S-transferase* (*VvGST*) gene, verified its function via CRISPR-mediated gene silencing combined with spray-induced gene silencing (SIGS) technology, and found that VvGST-silenced ‘Chardonnay’ grapevines exhibited superior physiological indicators compared to the control under severe water deficit, with enhanced drought resistance by activating the ABA signaling pathway and antioxidant system [208].

### 7.2. Multi-Omics and Live Imaging

Modern biotechnology increasingly relies on multi-omics approaches, including transcriptomics, metabolomics, epigenomics, proteomics, and integrated multi-omics analyses. These technologies can systematically decode the interaction networks of root signal modules and identify key regulatory nodes, directly addressing the ‘unclear mechanism’ issue in signal pathway crosstalk [209,210,211,212]. For instance, in response to N and/or P deficiency, researchers analyzed dwarf apple rootstock ‘M9-T337’ via multi-omics, which showed inhibited above-ground growth but optimized root traits, suppressed root NO_3_^−^ influx, and induced MdEXPA4/MdEXLB1, whose overexpression enhanced root development and N/P deficiency tolerance in transgenic plants [117]. Single-cell sequencing enables the characterization of signaling pathways across different cell types. For example, Zhang et al. used single-cell sequencing to identify key transcriptional regulators (e.g., the heat-sensitive ERF6 as a negative regulator), offering novel insights into somatic embryogenesis in longan at single-cell resolution [213]. In the single-cell RNA-seq studies of apple root tips, eight distinct tissue types have been identified, including epidermis (root hairs), root cap, exodermis, cortex, endodermis, xylem (vessels), vascular tissues, and stem cells, while cluster 16 and cluster 18 remain unclassified due to the lack of specific marker genes and limited functional characterization [214,215,216]. Fluorescence reporters and live imaging techniques have been employed to visualize real-time dynamics of signaling pathways, including hormones, Ca^2+^, and ROS [217,218,219]. Fluorescence imaging has been used to monitor lignin deposition in apple roots following pathogen infection [220].

#### Artificial Intelligence (AI)

AI are widely applied in plant research, which can deeply integrate multi-source data such as transcriptomics and phenomics, accurately realize dynamic monitoring of plant phenotypes, prediction of gene functions, and screening of stress-resistant varieties, greatly improving breeding efficiency and precision, and providing technical support for the high-yield and high-quality development of modern agriculture [221]. In plant phenomics research, AI enabled the development of an open-source deep learning tool named ‘Deep Plant Phenomics’, which provides pre-trained neural networks for common phenotyping tasks and an accessible platform for custom model training [222]. For example, a comparative study of eight machine learning models on 180 ‘Suncrest’ peach datasets showed that the artificial neural network (ANN) model achieved the best performance with an average AUC of 0.782 [223]. Ruiz-Muñoz et al. proposed a CNN-based super-resolution framework for plant root images (using FSRCNN and SRGAN architectures with three training strategies), which improved low-resolution root image quality, outperformed bicubic interpolation, and enhanced root-background segmentation performance [224]. TETRIS, a low-cost real-time chemical phenotyping system with screen-printed sensors, which monitors root environment salt, pH, H_2_O_2_, measures crop ion uptake, predicts via machine learning, and aids high-throughput screening for stress-resistant high-yield [225]. However, the uneven quality and incomplete nature of current training datasets limit the accuracy and generalizability of AI predictions, necessitating further refinement.

## 8. Limitations and Unknowns

### 8.1. Limitations

Several key issues restrict identification of signal pathways and application in fruit crops.

Firstly, different genotypes with contrasting tolerance to combined stressors exhibit distinct signaling patterns in citrus [226], apples [227], grapes [228], cherries, and pears [229]. The widespread use of grafting facilitates long-distance transmission of signals between roots and shoots, making it difficult to distinguish whether stress responses are autonomously initiated by roots or regulated by shoot feedback. Signals from different tissues are often tissue-specific and usually act synergistically to adapt to environmental changes. For instance, in root–shoot signal transduction, ET produced by roots under high-temperature stress reduces cytokinin levels in shoots, leading to premature leaf senescence [230].

Secondly, most fruit trees possess the biological characteristic of a long growth cycle, and their poor regeneration ability hinders the establishment of efficient transformation systems. This results in low transformation efficiency, which limits the predictability of field performance of laboratory-optimized traits under stress conditions. Meanwhile, genomic features of heterozygous genetic backgrounds and mostly polyploidy increase the difficulty of stable genetic transformation and genotype purification, restricting the accuracy of obtaining homozygous mutants for functional verification of epigenetic regulators and exacerbating the complexity in interpreting phenotype–genotype correlations [231].

In addition, the high cost and complexity of single-cell sequencing, along with the technical demands and marker limitations of live imaging, restrict their broad application in fruit crop root research.

Finally, gene editing technologies carry off-target risks, and transgenic-related safety concerns (e.g., marker gene residues, gene escape) have not been comprehensively and systematically evaluated.

### 8.2. Strategies

To overcome these limitations, targeted strategies are proposed to tackle the bottlenecks hindering signal pathway identification. For Agrobacterium-mediated transformation, whose efficiency is largely affected by explant type and hormone composition, optimizing strains [232], explants [233], co-culture conditions [234], vectors [235], and regeneration systems [236] serves as a core research direction in apple and citrus breeding. For example, the Root-borne Shoot (RBS) or high-pressure propagation breeding (HPPB) transformation technology overcomes the root-to-shoot conversion barrier in fruit crops and has been successfully applied to cherry and blueberry [19,185]. For gene editing, optimizing sgRNA design and adopting secondary regeneration tackle off-target risks (CRISPR/Cas9 is highly specific in grapevine [237]) and issues such as polyploid interference and T0 chimerism (70% in apple/pear) [238].

Multi-omics and live imaging technologies can assist in micrografting experiments, track the dynamic changes in graft-transmissible signals, and accurately dissect the signal transduction between roots and shoots.

### 8.3. Unknowns

Beyond experimental constraints, several key scientific unknowns remain to be addressed. The crosstalk mechanisms between different signaling pathways and epigenetic modifications in mediating root stress responses are not fully elucidated, particularly whether they act synergistically or antagonistically under complex field stresses. Additionally, the specificity of root signaling pathways and epigenetic modifications across different cell types and developmental stages lacks systematic characterization, despite initial insights from single-cell sequencing. The heritability of stress-induced epigenetic marks in fruit tree roots and their transgenerational regulatory roles also remain unclear. Furthermore, the interaction between transgenic root traits and rhizosphere microbiota, as well as the ecological implications of gene escape from transgenic roots, require in-depth assessment.

## Figures and Tables

**Table 1:** Second Messengers of aboitic stresses in fruit tree root.

Stressors	Concentration	Species	Sensitive Period	References
Ca^2+^	0.15~0.40 μmol/L	Grapevine	-	[80,81,83,84,85,86,87,88]
	0.1 mM	Apple	5 min	[82]
ROS	H_2_O_2_^−^ O_2_^−^ −	Apple	-	[93,94,95,96,97]
	H_2_O_2_ 100 mmol/L	Strawberry	13 d	[98]
	H_2_O_2_^−^	Grapevine	25 d	[99]
	H_2_O_2_ 10 mmol/L	Citrus	8 h	[107]
NO	50 μmol/L	Apple	6 d	[100]
	0.1 mmol/L	Strawberry	28 d	[103]
	15 μmol/L	Peach	10 min	[102]
	1 mmol/L	Banana	11 d	[101]
		100 μmol/L	Citrus	48 h	[110]
H_2_S	0.5 mmol/L	Apple	72 h	[97]
	200 μmol/L	Peach	4–72 h	[108,109]

“−” indicates that there is no specific concentration value for this ROS.

## Data Availability

No new data were created or analyzed in this study.
